# Changes in Biceps Femoris Long Head Fascicle Length after 10-d Bed Rest Assessed with Different Ultrasound Methods

**DOI:** 10.1249/MSS.0000000000002614

**Published:** 2021-01-21

**Authors:** FABIO SARTO, ELENA MONTI, BOŠTJAN ŠIMUNIČ, RADO PIŠOT, MARCO V. NARICI, MARTINO V. FRANCHI

**Affiliations:** 1 Department of Biomedical Sciences, University of Padova, Padova, ITALY; 2 Science and Research Centre Koper, Koper, SLOVENIA; 3 CIR-MYO Myology Center, University of Padova, Padova, ITALY

**Keywords:** HAMSTRINGS, MUSCLE ARCHITECTURE, EXTENDED FIELD OF VIEW, UNLOADING, INJURY PREVENTION

## Abstract

**Purpose:**

This study aimed to investigate the changes in fascicle length (Lf) of biceps femoris long head (BFlh) after 10 d of bed rest (BR) by comparing four different ultrasound (US) methods.

**Methods:**

Ten healthy men participated in 10-d BR. Before (BR0) and after (BR10) the BR period, BFlh Lf values were obtained using 1) extended-field-of-view (EFOV) technique, 2) the manual linear extrapolation (MLE) method, and 3) two trigonometric equations (equations A and B) from a single US image.

**Results:**

After BR10, decreased Lf values were observed by EFOV (*P* < 0.001; Hedges’ *g* = 0.29) and MLE (*P* = 0.0082; *g* = 0.22) methods, but not with equations A and B. Differences between equation A and the other US methods were detected at both time points. The percentage of changes in Lf between BR0 and BR10 was influenced by the US methods applied, with difference detected between the changes measured by EFOV and the ones estimated by equation A (*P* = 0.04; *g* = 0.53). Bland–Altman analyses revealed relevant average absolute biases in Lf between EFOV and other methods at both time points (range BR0–BR10: MLE, 0.3–0.37 cm (3.4%–4.32%); equation B, 0.3–0.48 cm (3.24%–5.41%); equation A, 2.44–2.97 cm (24.05%–29.2%)). A significant correlation (*r* = 0.83) in percentage of change in Lf values was observed only between EFOV and MLE.

**Conclusions:**

We showed that four distinct US methods lead to different results in the assessment of BFlh Lf changes after a short-term period of unloading. The implementation of EFOV technique (or alternatively MLE) to assess Lf changes in BFlh during longitudinal studies is warranted.

Biceps femoris long head (BFlh) fascicle length (Lf) has been commonly assessed *in vivo* using ultrasound (US) imaging techniques (1–3) and has been associated with hamstrings strain injury risk (4) and eccentric strength (5). For this reason, previous investigations have studied the architectural adaptations of BFlh to concentric (6,7) and eccentric training (6–16), sprint training (17), and stretching interventions (18). Conversely, only few studies investigated BFlh architectural remodeling after pure unloading (19) and detraining (7,12–14), mostly carried out successively to training periods consisting of different modalities of eccentric exercise (i.e., from isokinetic dynamometer training to Nordic hamstring exercise and its variations). In contrast to training studies, the detraining literature shows that, after 2 to 4 wk of detraining, marked reductions in BFlh Lf occur, associated with a relevant increase in pennation angle (PA) and slight or no changes in muscle thickness (MT) (7,12–14).

However, the assessment of BFlh Lf is not easy, as BFlh exhibits a complex muscle morphology (20), accompanied by a heterogeneous architectural arrangement of its fascicles, which appears nonuniform at different portions of its length (21,22). Furthermore, one limitation of most studies using US imaging for the assessment of BFlh Lf derives from the small field of view (FOV) of nearly all US linear transducers when compared with the total length of the fascicles, thus exposing the pitfalls of extrapolation methods (3). Such pitfalls are represented by the fact that fascicles and aponeuroses curvatures are generally neglected by extrapolation methods, and that muscle architecture is assumed to be homogenous along the entire muscle length (3). The four most common methods used for the assessment of BFlh Lf from US images are as follows: 1) the manual linear extrapolation (MLE) method, which consists of extrapolating visible fascicles with straight lines over the nonvisible portion of the muscle up to the intersection point with the linearly-projected superficial aponeurosis of the muscle (9); 2) a trigonometric equation in which the whole Lf estimation is based on the variables MT, PA, and the angle between the aponeuroses (this formula will be termed as “equation A”—for a description, please see the Methods section) (23); 3) a second trigonometric equation in which only the nonvisible portion of the fascicle is linearly extrapolated (this will be called “equation B”) (24); and 4) the extended-FOV (EFOV) US method, which allow us to measure Lf directly by means of a panoramic scan without any extrapolation needed (25). When comparing single snapshots of US images and EFOV scans (both acquired at rest and at the same region of interest (ROI)), a greater Lf was found when using equation B compared with the digitization of the full visible fascicle (absolute error between 0.74 and 0.93 cm) (2). Another recent study (3) showed that both trigonometric equations A and B lead to an overestimation of Lf values compared with the ones obtained by the EFOV method in the same BFlh ROI of the volunteers. Particularly, equation A resulted in a Lf overestimation of almost ~2 cm: nevertheless, such error seems to be not systematic, as Bland–Altman analyses revealed the presence of both underestimation and overestimation cases (although the tendency of overestimation was greater) (3).

Interestingly, these observations have only been reported for a single time point in studies conducted with a cross-sectional design; therefore, to date, no data are available concerning the possibility that these methodological issues regarding Lf extrapolation could affect the results of longitudinal studies.

For this reason, we investigated the Lf changes in BFlh after a short period of complete unloading, comparing the four different US methods explained previously. A bed rest (BR) study design was chosen in order to reproduce a full unloading condition that could mimic the hospitalization condition experienced after injuries and is likely to lead to more accentuated architectural adaptations compared with other disuse models (i.e., detraining, unilateral limb suspension).

We hypothesized that different methods would lead to different results in the magnitude of changes of BFlh Lf in response to BR.

## METHODS

### Participants

Ten healthy men (age, 22.9 ± 5 yr; body mass, 77.5 ± 10 kg; height, 1.81 ± 0.04 m) took part in a 10-d BR study. All subjects passed kinesiological and medical examination, physical activity questionnaire (Global Physical Activity Questionnaire), body composition analysis, resting and exercise electrocardiography with blood pressure assessment, medical questionnaires, functional movement assessment, and nutrition interview. Inclusion criteria were as follows: 20–32 yr, 21 < body mass index <28 kg·m^−2^, moderate to vigorous physical activity >90 min·d^−1^, normal electrocardiography and blood pressure, functional movement score <18, and muscle mass >16%. Exclusion criteria were acute or chronic skeletal, neuromuscular, metabolic and cardiovascular disease conditions, and pulmonary embolism.

The participants were housed in standard air-conditioned hospital rooms and were under constant visual surveillance with 24-h medical care, 24-h heart rate, and physical activity monitors. During BR, the subjects performed all daily activities in bed with no deviations from the horizontal lying position permitted, and both exercise and muscle contraction tests were not allowed in the 10-d period. The study was conducted in accordance with the declaration of Helsinki and was approved by the Slovenian National Medical Ethics Committee. All volunteers were fully informed of the procedures and gave written, informed consent. The project was carried out in August and September 2019 in the Izola General Hospital (Izola, Slovenia).

### US imaging

**FIGURE 1:**
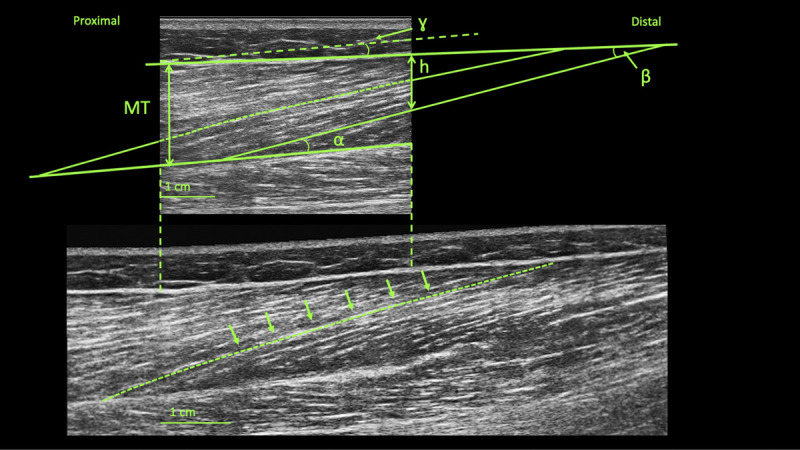
B-mode snapshot (upper image) and EFOV (lower image) scans obtained from a representative subject. α, PA; γ, angle between aponeuroses; β, the angle between the linear projection of the fascicle and the linear extension of the superficial aponeurosis; *h*, linear distance between the superficial aponeurosis and the end point of the fascicle.

Before (BR0) and after (BR10) the BR period, BFlh muscle images were obtained using B-mode ultrasonography (Mylab 70; Esaote Biomedica, Genova, Italy), using a linear 4.7-cm probe. At BR0, scans were collected ~30 min after the beginning of the BR, whereas at BR10, scans were obtained just before the standing up. For the analysis of the reliability, US images were acquired also 2 d before BR0 (BDC1). The methods relative to the US procedures have been described previously (3). Briefly, the subjects were asked to lie down prone on the bed and to relax during the acquisition of the scans. The 50% of the femur length was marked at mid-distance between great trochanter and midpatella point. Markers’ visibility was checked at the end of each day, and the same tracing was marked again in order to ensure its visibility during image acquisition throughout the whole BR period. The images were obtained with the transducer positioned at 50% of femur length in the mid of the muscle belly (detected as the midpoint between the medial and lateral borders via US), maintaining the superficial and the intermediate aponeurosis parallel to each other. Scans of a representative subject are presented in Figure 1.

For the acquisition of the longitudinal EFOV images, the transducer was placed parallel to the fascicle plane and then moved slowly and without interruptions from the 70% to 30% of the BFlh length (i.e., proximal to distal). The selected path was followed adjusting the transducer orientation to stay in the fascicle plane (2,3). Two B-mode and two EFOV images for each subject were collected at each time point.

Transverse EFOV images were also collected for the assessment of a single BFlh cross-sectional area (CSA) collected at the 50% of the total femur length, as previously described in details (26). Briefly, the 50% of the distance between the greater trochanter and the midpatellar point was detected and marked. In the same ROI, the transducer was placed on the lateral portion of the posterior thigh and then moved on the transversal plane in a lateral-to-medial fashion. A sufficient amount of transmission gel was applied to the transducer for all scans.

### Muscle architecture and CSA assessment

Because the inclination of the aponeurosis is considered an important factor that can influence the architectural outcomes (23,27), all the analyzed images had the angle between the superficial and intermediate aponeurosis lower than 4° (3). For the analyses of Lf, two inclusion criteria were considered: i) the clear visibility of fascicle insertion into the intermediate aponeurosis and ii) the visibility within the ROI of a fascicle portion representing at least 25% of the estimation of the total length (3). One B-mode and one EFOV scan were analyzed both at BR0 and BR10. In B-mode scans, Lf was estimated with three different methods: 1) MLE, 2) equation A, and 3) equation B. The MLE method was applied to measure with the segmented line tool the visible portion of the fascicle and then extrapolating it with a straight line until the extension of the superficial aponeurosis (9).

For the second method, Lf was computed using the following trigonometric equation A (23):


$$\mathrm{Lf}=\sin\left(\gamma +90{}^{\circ}\right)\times \mathrm{MT}/\sin\left(180{}^{\circ}-\left(\gamma +180{}^{\circ}-\theta \right)\right)$$ [A]

where γ is the angle between the superficial and the intermediate aponeurosis, MT is the averaged MT, and *θ* is the PA in degrees.

In the third method, Lf was estimated using trigonometric equation B (24):


$$\mathrm{Lf}=L+\left(h/\text{sin β}\right)$$ [B]

where *L* is the visible Lf in the ROI of the image, *h* is the linear distance between the superficial aponeurosis and the end point of the fascicle, and β is the angle, in degrees, between the linear projection of the fascicle and the linear extension of the superficial aponeurosis.

For the EFOV images, the ROI corresponding to the one analyzed in the single snapshot images was identified by mean of the previously measured length of the femur. Afterward, Lf was directly measured with the segmented tool because every single fascicle included in the analysis laid within the ROI. In case the fascicle was not continuously visible, care was taken in following its path in order to accurately estimate fascicle behavior. In average, three or four fascicles were included in the analysis of each image and method.

Lf, PA, and MT were assessed using ImageJ software (1.52v; National Institutes of Health, Bethesda, MD). PA was calculated as the insertion angle between the fascicle and the intermediate aponeurosis. MT was measured as the linear distance between the two aponeuroses. The average of three different MT measured within the ROI was used for the data analysis. CSA values were calculated by tracing the contours of BFlh using ImageJ software (1.52v; National Institutes of Health).

### Statistical analysis

Descriptive statistics have been reported as mean and SD. Normality of data was assessed through qualitative visual inspection using QQ plot, skewness and kurtosis calculation, and Shapiro–Wilk normality test. Inferential statistics have been presented as point estimates and corresponding confidence intervals (95% CI). Reliability of the measurements was tested for Lf values via calculation of the intraclass correlation coefficient (ICC) and coefficient of variation of the standard error mean (CV SEM) on five subjects, comparing scans at BDC1 and BR0. For the ICC calculation, a two-way mixed-effect, absolute agreement test was used, following the criteria by Koo and Li (28). In addition, absolute and percentage minimum detectable change (MDC; i.e., the minimum difference suggesting a real change, which would not be dictated by repeated measurements errors) was calculated, following the approach suggested by Weir (29). Paired *t*-tests were carried out to detect differences in Lf (for each method: EFOV, linear extrapolation, equation A, and equation B), PA, MT, and CSA before and after BR period. One-way repeated-measures ANOVA was performed both at BR0 and BR10 to identify differences between techniques on Lf values. Bonferroni *post hoc* tests were performed to determine if the methods significantly differ from each other. The same statistical analysis and *post hoc* test were carried out to compare the differences in Lf pre-to-post changes (absolute delta changes) between all techniques. Correlations were tested using the Pearson’s product moment correlation coefficient (*r*). The agreement between techniques was assessed using Bland–Altman analyses at both time points (30). The magnitudes of changes were also expressed as standardized mean difference Hedges’ *g*, computed as follows:


$$g=\frac{x_{1}-x_{2}\;}{s^{*}}\;\left(1-\frac{3}{4\left(n_{\text{pairs}}-1\right)-9}\right)$$

where *x_1_* − *x_2_* is the difference between means of the two groups, *n*_pairs_ is the is the number of pairs, s* is the pooled SD calculated as:


$$s^{*}=\sqrt{\frac{s_{1}^{2}+s_{2}^{2}}{2}}$$

with *s*_1_ and *s*_2_ representing the SD of each group.

Hedges’ *g* was interpreted as trivial (*g* ≥ 0.19), small (0.2 ≤ *g* ≤ 0.49), medium (0.50 ≤ *g* ≤ 0.79), and large (*g* ≥ 0.80) effects (31). The level of significance was set at *P* < 0.05. Data analysis was performed using GraphPad Prism software (version 8.00; GraphPad Software, San Diego CA).

## RESULTS

**FIGURE 2:**
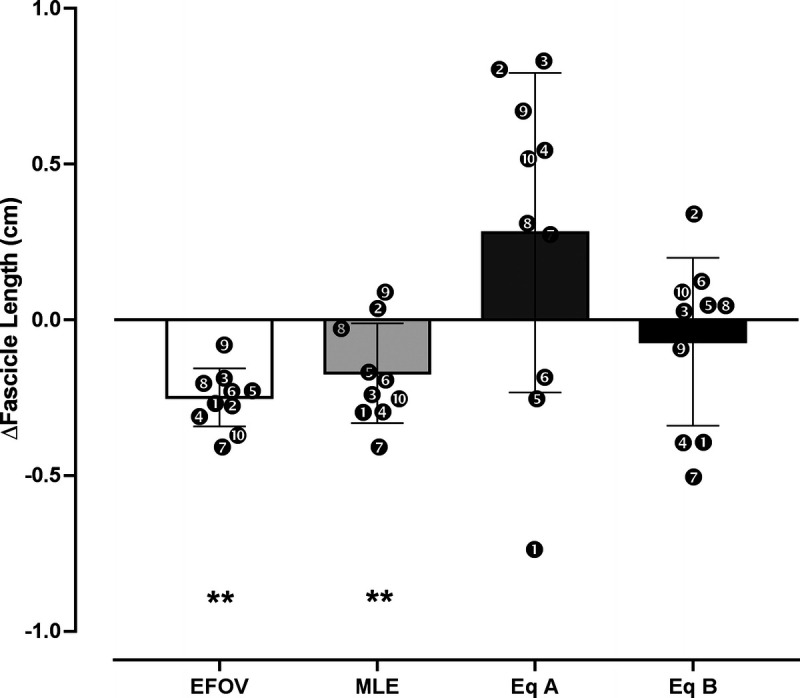
Comparison of absolute changes (in centimeters) in Lf values between BR0 and BR10 assessed by extrapolation methods (MLE, equation A, and equation B) and EFOV scans. **P < 0.01.

The values of all the parameters necessary for Lf estimation and the CSA values are presented in Table 1, whereas absolute vales in Lf at BR0 and BR10 are presented in Table 2. The ICC, CV SEM, and MDC for Lf values of each method are illustrated in Table 3. Differences in Lf values between BR0 and BR10 were observed for EFOV (*P* < 0.001; *g* = 0.29 (95% CI, −0.32 to 0.85); Δ = −0.25 cm (−2.84%)) and MLE (*P* = 0.0082; *g* = 0.22 (95% CI, −0.4 to 0.86); Δ = −0.17 cm (−1.94%)), but not for equations A (*P* = 0.1176; *g* = 0.19 (95% CI, −0.84 to 0.41); Δ = +0.28 cm (+2.52%)) and B (*P* = 0.4327; *g* = 0.09 (95% CI, −0.53 to 0.72); Δ = −0.07 cm (−0.85%); Fig. 2). No changes over this period were detected in PA and MT, whereas CSA was significantly reduced (*P* = 0.02; *g* = 0.22 (95% CI, −0.4 to 0.86); Table 1). The Lf values were significantly influenced by the US techniques used at BR0 (*P* < 0.001) and at BR10 (*P* < 0.001). *Post hoc* analysis showed differences between the EFOV technique and equation A at both time points (BR0: *P* < 0.001, *g* = 2.14 (95% CI, 1.1 to 3.64); BR10: *P* < 0.001 *g* = 2.47 (95% CI, 1.3 to 4.08)), and EFOV technique and equation B at BR10 (*P* = 0.03; *g* = 0.55 (95% CI, 0.09 to 1.26)). Lf values estimated with MLE differed from those calculated with equation A both at BR0 (*P* = 0.0018, *g* = 1.93 (95% CI, 0.95 to 3.33)) and at BR10 (*P* = 0.0011, *g* = 2.18 (95% CI, 1.1 to 3.64)). Moreover, equation A led to a systematic bias of the Lf values compared with equation B both at BR0 (*P* = 0.0024; *g* = 1.98 (95% CI, 0.96 to 3.34)) and BR10 (*P* < 0.001; *g* = 2.07 (95% CI, 1.03 to 3.47)).

**TABLE 1:** Muscle CSA values obtained by transversal EFOV scans acquired at 50% of femur length and parameters of muscle architecture used to for estimation of Lf obtained from B-mode snapshot scans at BR0 and BR10.

	BR0, Mean (SD)	BR10, Mean (SD)	Δ Values, Mean (95% CI)	Δ%, Mean (95% CI)	*P*
CSA, cm^2^	11.62 (2.11)	11.11 (1.93)	−0.51 (−0.89 to −0.13)	−4.25 (−7.55 to −0.95)	0.02*
MT, cm	2.15 (0.29)	2.11 (0.32)	−0.04 (−0.11 to 0.03)	−1.89 (−4.95 to 1.17)	0.28
PA, °	12.74 (0.91)	12.67 (0.86)	−0.07 (−0.26 to 0.09)	−0.54 (−1.73 to 0.72)	0.32
γ (equation A), °	1.73 (0.68)	2.11 (1.22)	+0.38 (−0.22 to 0.98)	+19.89 (−26.9 to 66.7)	0.24
β (equation B), °	13.16 (1.16)	13.6 (0.82)	+0.44 (−0.08 to 0.96)	+3.67 (−0.38 to 7.72)	0.13
*h* (equation B), cm	1.07 (0.23)	1.03 (0.21)	−0.05 (−0.05 to 0.15)	−3.18 (−7.22 to 13.6)	0.37
% visible Lf of total Lf	49.13 (5.25)	52.50 (7.34)	+3.38 (−1.12 to 7.88)	+6.44 (−1.33 to 16.1)	0.17

γ, angle between aponeuroses, β, the angle between the linear projection of the fascicle and the linear extension of the superficial aponeurosis; *h*, linear distance between the superficial aponeurosis and the end point of the fascicle; Δ values, absolute change between BR0 and BR10; Δ%, percentage of change between BR0 and BR10.

*P < 0.05.

**TABLE 2:** Lf differences in absolute values at BR0 and BR10 for each US technique.

	Lf EFOV, cm	Lf MLE, cm	Lf Equation A, cm	Lf Equation B, cm
BR0	8.82 (0.76)**	9.12 (0.75)*	11.26 (1.23)	9.12 (0.65)*
BR10	8.57 (0.59)**^,^ ***	8.95 (0.76)*	11.54 (1.33)	9.05 (0.80)**
BR0 vs BR10, *P* value	<0.001	0.0082	0.1176	0.4327

Values are presented as mean and standard deviation (SD).

*P < 0.01 versus equation A within the same time point.

**P < 0.001 versus equation A within the same time point.

***P < 0.05 versus equation B within the same time point.

**TABLE 3:** ICC, CM SEM, MDC, and MDC% for Lf values of each US technique.

	EFOV	MLE	Equation A	Equation B
ICC	0.97	0.96	0.84	0.87
SEM	0.09	0.09	0.18	0.16
CV SEM%	1.02	1.03	0.17	0.20
MDC, cm	0.24	0.25	0.5	0.46
MDC%	2.78	2.73	4.85	4.99

**FIGURE 3:**
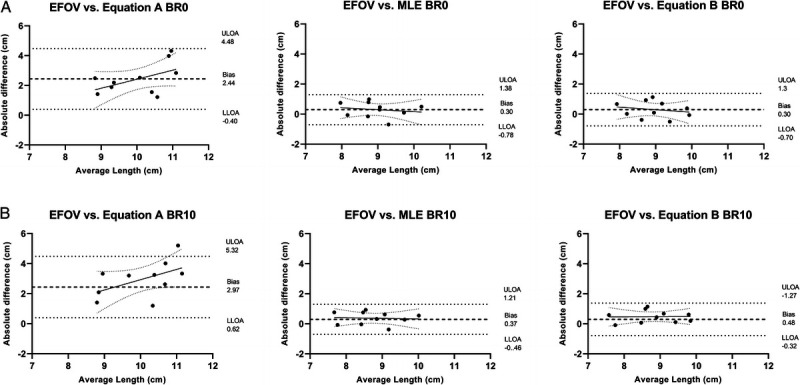
Agreement of Lf measurements between EFOV and extrapolation methods (MLE, equation A, and equation B): Bland–Altman analyses showing absolute differences with respect to the average Lf obtained between methodologies at BR0 (A) and BR10 (B). LLOA, lower limit of agreement; ULOA, upper limit of agreement.

In addition, the percentage of changes in Lf values between BR0 and BR10 was influenced (*P* = 0.0067) by the US techniques applied. *Post hoc* analysis detected a difference between the changes measured by EFOV and the ones estimated by equation A (*P* = 0.04; *g* = 0.53 (95% CI, 0.13 to 1.27)). A significant correlation between EFOV technique and MLE method percentage of changes in Lf values was observed (*P* = 0.0033; *r* = 0.83 (95% CI, 0.41–0.96)), but not for the trigonometric equations (equation A: *r* = 0.05 (95% CI, −0.6 to 0.66); equation B: *r* = 0.50 (95% CI, 0.19 to 0.86)). Bland–Altman plots at BR0 (Fig. 3A for absolute values) revealed average absolute biases in Lf between EFOV technique and equation A (2.44 ± 1.04 cm; 24.05% ± 9.02%), equation B (0.3 ± 0.56 cm; 3.24% ± 6.24%), and MLE (0.3 ± 0.51 cm; 3.4% ± 5.79%). Moreover, at BR10 (Fig. 3B), the average absolute biases in Lf compared with the EFOV technique were of 2.97 ± 1.2 cm (29.2% ± 10.6%) for equation A, 0.48 ± 0.41 cm (5.41% ± 4.75%) for equation B, and 0.37 ± 0.43 cm (4.32% ± 5.03%) for MLE.

## DISCUSSION

With the present study, we aimed to investigate whether the methodological limitations of BFlh Lf extrapolation, previously observed in cross-sectional studies (2,3), may also affect the assessment of Lf changes in a longitudinal study design. To this end, we assessed the BFlh Lf changes in response to short-term BR using four different US methods. The main findings were the following: (i) evidence of a reduction in BFlh Lf after 10 d of BR was only provided by the EFOV technique and MLE method; (ii) the Lf values were influenced by the US methods used both at BR0 and BR10; and (iii) a significant correlation in percentage of change in Lf values was observed only between EFOV and MLE.

BR resulted in a significant reduction in BFlh muscle size, in accordance with previous works (19,32) (Table 1). Our findings confirm the notion that BFlh, being not a postural muscle, undergoes a relatively mild atrophy (−4.25%) in response to unloading (33). To date, however, no previous study investigated the adaptations of BFlh Lf to unloading. After 10 d of complete unloading, significant changes in BFlh Lf were only observed when using the EFOV technique and MLE method, although these changes were of a small magnitude. Conversely, no differences could be detected using either of these trigonometric equations.

Our results highlighted that the BFlh Lf extrapolation pitfalls (2,3) may influence the findings of longitudinal studies. These observations are relevant from a clinical and training perspective because the choice of the US method used may be crucial for the detection of potential changes after training/detraining interventions. Interestingly, we found only little changes (MLE: −0.17 cm (−1.94%); EFOV: −0.25 cm (−2.84%)) in Lf after 10 d of BR. The mean absolute and percentage difference for EFOV was just higher than the calculated MDC (0.24 cm; 2.77%), whereas for MLE, it was lower than its MDC (0.25 cm; 2.73%; Table 3). Both with EFOV and MLE analysis, half of the subjects presented decrements in Lf that were higher than their respective MDC. These results are in contrast with previous findings of the changes in BFlh Lf during detraining/training cessation, which showed that even short detraining periods (2–4 wk) are sufficient to induce large decreases in Lf (average range, −0.93 to −2.5 cm) (12–14). When comparing these previous studies with the present one, it is important to consider that pure unloading and detraining (i.e., training cessation) are distinct models of muscle disuse. In fact, although BR is well known to induce marked muscle atrophy (32,33) and architectural adaptations (33), in studies that have investigated changes in BFlh Lf due to detraining, the observed muscle loss could be affected by the preceding training intervention, potentially accelerating such process (34). However, it must be considered that, in detraining studies, the volunteers are usually allowed to perform habitual sports activity (i.e., practically they just refrain from performing the specific exercise previously investigated) (7,12–14). In addition, the architectural adaptations observed for other muscle groups during detraining are generally of smaller magnitude than the one observed for BFlh. For instance, no or little reductions in vastus lateralis Lf were found in detraining periods of 4–14 wk (35–37). Such inconsistencies may be related to either the possible singular behavior of BFlh muscle during detraining or to the US analysis method used in previous investigations (i. e., equation A).

Our results also suggest that the US methods used influenced the Lf values obtained both at BR0 and BR10, similarly to the findings previously obtained at a single time point (2,3). Indeed, compared with EFOV, relevant differences in absolute Lf values were observed (range BR0–BR10: MLE, 0.3–0.37 cm (3.4%–4.32%); equation B, 0.3–0.48 cm (3.24%–5.41%); equation A, 2.44–2.97 cm (24.05%–29.2%)). The main issues regarding BFlh Lf assessment at rest obtained by extrapolation methods are that (i) BFlh has a heterogeneous fascicle organization along its length (21,22); (ii) some fascicles may present a characteristic curvature, resulting in a “S” shape (2,3); and (iii) aponeuroses are not linear outside the FOV; thus, the curvature is neglected (3). Among the extrapolation methods analyzed in this study, Lf values obtained with equation A exhibited the largest differences with the ones measured by EFOV. Moreover, no correlation was found between percentages of changes in Lf values assessed by EFOV and those obtained with the two trigonometric methods. Equation A is computed only with MT, PA, and the angle between aponeuroses (γ); thus, the final estimation of Lf values is strictly dependent on those parameters, as no visible portion of the fascicle is digitized. This extrapolation method has been validated for vastus lateralis (a muscle with consistent fascicle curvature) but not the vastus medialis because of its regionally variable and considerable fascicle curvature (23). Little or no changes have been observed in BFlh MT in response to training (6,7,11–13), detraining (7,12–14), and unloading (in our present study). Thus, in longitudinal studies, changes assessed with equation A mainly rely on PA measurement. In the present study, no changes in PA were detected after 10 d of unloading, and this was somehow unexpected, as a reduction in BFlh PA has been observed in another BR investigation (19). Conversely, an increase in BFlh PA is generally observed after periods of detraining (7,12–14). This behavior of fascicle geometrical rearrangement is surprising because unloading and detraining scenarios are generally associated with a decrease in PA (38), and such increase in PA has never been observed in any other muscle group. This peculiar increase in PA may explain the drastic decrease in BFlh Lf detected in detraining studies when equation A was used. Future studies should further explore the BFlh PA in response to unloading/detraining stimuli.

In case also PA remains constant after an intervention (as observed in the present study), Lf values measured with equation A would depend almost exclusively on the change of the angle between aponeuroses (γ). This angle may be influenced by individual anatomical changes or methodological US-related factors (e.g., transducer tilt and alignment during image acquisition), or a combination of both. Our results suggest that, in the absence of MT and PA adaptations, even small changes in this parameter could lead to relevant differences in Lf estimation. For example, in one volunteer, after the BR period, although similar MT (2.54 cm both at BR0 and BR10) and PA (14.37° at BR0 vs 14.19° at BR10) were maintained, a small increase in γ (1.43° at BR0 vs 1.99° at BR10) resulted in a considerable increase of 0.67 cm in Lf (11.34 cm at BR0 vs 12.01 cm at BR10) when calculated with equation A. Although the changes of the angle between aponeuroses may play an important role in the Lf estimation using equation A, the absolute γ preintervention and postintervention values are generally not reported in previous works. We advise that future studies should report γ values and further investigate the influence of this parameter on Lf estimation, potentially identifying cutoff values for scan inclusion–exclusion criteria.

Furthermore, the analysis of muscle architecture could be associated with variability related to the manual digitalization process. In this regard, automated analysis could improve the reliability of such measurements (particularly when multiple raters are involved) by removing the variability induced by manual segmentation (39). For this scope, a new ImageJ macro tool to automate measurements in B-mode US scans has been proposed recently by Seynnes and Cronin (39). However, this tool relies on muscle fascicle orientation and thus may be not yet suitable for muscles that present complex and variable muscle architecture arrangement, such as BFLh. Therefore, we cannot exclude that manual digitalization variability could have influenced our results. Future work should focus on the implementations of new automatic tools fit for the analysis of BFlh muscle architecture.

Interestingly, MLE displayed the smallest mean absolute bias (Fig. 3) compared with EFOV values. In addition, a strong correlation (*r* = 0.83; 95% CI, 0.41–0.96) was found in the Lf percentage of changes only between the EFOV technique and MLE method. These findings may suggest that, at least in longitudinal studies where limited changes in Lf occur, the MLE and EFOV exhibit an excellent agreement. The main advantage of MLE over trigonometric equation A is that the visible portion fascicle is directly digitized inside the FOV. Although visible portions of the BFlh fascicles were only the ~50% of their total resting length (assessed with MLE method; Table 1) (2,3), the accuracy of extrapolation methods compared with the EFOV technique is probably dependent on the length of the fascicle that is visible and on the FOV (1). Noteworthy, in this study, we used a linear transducer of 4.7 cm; therefore, the agreement between the EFOV technique and MLE method and may further increase with a larger FOVs (1).

This study has some limitations. First, although the EFOV technique has the clear advantage of enabling measurements of the entire Lf, it should not be considered as the “gold standard” for the assessment of Lf. Intrinsic limitations of the EFOV technique have already been described elsewhere (3,27). BFlh architectural parameters measured by EFOV have been recently validated against cadaver specimens (22). However, in that study, more panoramic scans were collected at distinct muscle regions (30%, 50%, 70%, and 90% of the total muscle length), differently from our work in which a single image was acquired. We acknowledge that our observations are based on a small sample size; however, BR studies are considerably expensive and require a remarkable organization effort, so it was unrealistic to recruit more volunteers.

## CONCLUSIONS

This present work shows that different US methods lead to different results in the assessment of BFlh Lf changes after a short-term period of unloading. We recommend the implementation of EFOV scans to accurately assess Lf changes in BFlh during longitudinal studies. The MLE method with large FOV should be used if only conventional B-mode US imaging is available.
